# Characterization of Antimicrobial Resistance Patterns and Detection of Virulence Genes in *Campylobacter* Isolates in Italy

**DOI:** 10.3390/s140203308

**Published:** 2014-02-19

**Authors:** Elisabetta Di Giannatale, Gabriella Di Serafino, Katiuscia Zilli, Alessandra Alessiani, Lorena Sacchini, Giuliano Garofolo, Giuseppe Aprea, Francesca Marotta

**Affiliations:** LNR *Campylobacter*, IZSAM G.Caporale, via Campo Boario 64100 Teramo, Italy; E-Mails: g.diserafino@izs.it (G.D.S.); k.zilli@izs.it (K.Z.); a.alessiani@izs.it (A.A.); lo.sacchini@izs.it (L.S.); g.garofolo@izs.it (G.G.); g.aprea@izs.it (G.A.); f.marotta@izs.it (F.M.)

**Keywords:** antimicrobial susceptibility, *Campylobacter*, microarray, PFGE, sequencing, virulence

## Abstract

*Campylobacter* has developed resistance to several antimicrobial agents over the years, including macrolides, quinolones and fluoroquinolones, becoming a significant public health hazard. A total of 145 strains derived from raw milk, chicken faeces, chicken carcasses, cattle faeces and human faeces collected from various Italian regions, were screened for antimicrobial susceptibility, molecular characterization (SmaI pulsed-field gel electrophoresis) and detection of virulence genes (sequencing and DNA microarray analysis). The prevalence of *C. jejuni* and *C. coli* was 62.75% and 37.24% respectively. Antimicrobial susceptibility revealed a high level of resistance for ciprofloxacin (62.76%), tetracycline (55.86%) and nalidixic acid (55.17%). Genotyping of *Campylobacter* isolates using PFGE revealed a total of 86 unique SmaI patterns. Virulence gene profiles were determined using a new microbial diagnostic microarray composed of 70-mer oligonucleotide probes targeting genes implicated in *Campylobacter* pathogenicity. Correspondence between PFGE and microarray clusters was observed. Comparisons of PFGE and virulence profiles reflected the high genetic diversity of the strains examined, leading us to speculate different degrees of pathogenicity inside *Campylobacter* populations.

## 1. Introduction

*Campylobacter* is among the major causes of food-borne illness worldwide [1]. More than 200,000 confirmed cases of *Campylobacter* infections were reported in 24 member states of the European Union with a rate of 45.2 cases per 100,000 people, representing an issue of considerable socio-economic impact [2,3]. In addition to gastrointestinal forms, 1% of cases may develop peripheral neuropathies including Guillain-Barré syndrome (GBS), reactive arthritis (ReA) and functional bowel diseases, such as irritable bowel syndrome (IBS) [4,5]. One of the most common causes of post- infectious IBS in the United Kingdom is related to *Camplylobacter jejuni* [5]. The consumption of undercooked poultry meat and cross-contamination are leading risk factors for human campylobacteriosis [6]. The epidemiology of *Campylobacter* is complicated by the wide distribution of the bacterium and its genetic variability. *C. jejuni* is the most significant species associated with human infections [2]. A recent survey undertaken for estimating *Campylobacter* prevalence in chickens confirmed this pathogen as the most common zoonotic agent deriving from broiler flocks and broiler carcasses in Italy (72.3%) [7]. The increased bacterial resistance to antibiotics is a matter of special concern, representing a significant public health problem. *Campylobacter* has developed resistance to several antimicrobial agents over the years, including macrolides, quinolones and fluoroquinolones. In Italy, *Campylobacter* strains showed high resistance values to ciprofloxacin, tetracycline, nalidixic acid and erythromycin, while no resistance to gentamicin was observed [8]. Resistance to quinolones and fluoroquinolones is often related to spontaneous point mutations of target enzymes, resulting in substitution of aminoacids with the gyrase and topoisomerase genes (*gyrA*, *gyrB*, *parC*, *parE*). In gram-negative bacteria, *gyrA* mutations are correlated with quinolone and fluoroquinolone resistance and in particular in *Campylobacter* they are associated with substitution of threonine with isoleucine in position 86 [9,10]. No *gyrB* mutations have been reported for *Campylobacter* [11,12]. Despite the fact that campylobacteriosis is a leading food-borne disease in many developed countries, investigators are still at the initial stages of defining the genetic and phenotypic features responsible for its pathogenesis. In order to gain more information, all *Campylobacter* strains were assayed for antimicrobial resistance patterns and screened for virulence-associated genes involved in motility, adherence, invasion, toxin production, capsule synthesis and chicken gastro intestinal tract colonization. The aim of this study was to investigate the genetic diversity among strains of *C. jejuni* and *C. coli* of different origin to provide a model of laboratory surveillance network, where PFGE and microarray could contribute to recognize epidemic clones with a nationwide spreading pattern and with peculiar properties of virulence/antibiotic resistance.

## 2. Experimental Section

### 2.1. Bacterial Strain Collection

A total of 145 *Campylobacter* strains isolated from raw milk, chicken carcasses, chicken and cattle faeces and human stools were analysed (Table 1). *Campylobacter* from chickens were collected during 2008–2009 from various Italian regions (Piemonte, Lombardia, Veneto, Marche, Abruzzo and Campania) while *Campylobacter* from cattles were isolated during 2010–2011 in Piemonte region. *Campylobacter* from diarrhoeic human stools were isolated from three patients in Marche region in 2008 and from one patient in Abruzzo region in 2009. The strains were cultured on Columbia blood agar in microaerobic atmosphere at 42 °C and stored at −80 °C in Microbank™ until further analysis.

### 2.2. DNA Extraction and Polymerase Chain Reaction (PCR)

The strains were identified by multiplex PCR as described by Wang [13]. Strains used as positive controls were *C. coli* NCTC 11353; *C. fetus* ATCC 19438; *C. jejuni* ATCC 33291; *C. upsaliensis* NCTC 11541 and *C. lari* NCTC 11552. DNA was extracted using Ultraclean microbial DNA kit (*Mo Bio* Laboratories, Solana Beach, CA, USA) according to the manufacturer's instructions and quantified using a Nanodrop Spectrophotometer (Nanodrop Technologies, Celbio Srl., Milan, Italy).

PCR amplification was performed in 50 μL volumes containing 25 μL PCR Master Mix 2X (Promega Corporation, Madison, WI, USA), 25 mM MgCl2, 0.5 μM *C. jejuni* and *C. lari* primers; 1 μM *C. coli* and *C. fetus* primers, 2 μM *C. upsaliensis* primers 1 ng of genomic DNA/μL. DNA amplification was carried out in a DNA thermal cycler 9700 Applied Biosystems (Applied Biosystems, Foster City, CA, USA) following the steps indicated by Wang [13]. PCR products were analysed on 1.5% agarose gels, stained with Sybr Safe DNA gel (Invitrogen, Carlsbad, CA, USA) and photographed at the transilluminator (Alpha Innotech, San Leandro, CA, USA).

### 2.3. Antimicrobial Susceptibility

*Campylobacter* strains susceptibility to antibiotics was evaluated with the microdilution method using the Sensititre automated system (TREK Diagnostic Systems, Cleveland, OH, USA). Colonies were harvested on Columbia agar for 24 h and then seeded in Mueller Hinton Broth supplemented with blood and dispensed into Eucamp microtiter plates (TREK Diagnostic Systems), containing known scalar concentrations of the following antibiotics: gentamicin (0.12–16 μg/mL), streptomycin (1–16 μg/mL), ciprofloxacin (0.06–4 μg/mL), tetracycline (0.25–16 μg/mL), erythromycin (0.5–32 μg/mL), nalidixic acid (2–64 μg/mL), and chloramphenicol (2–32 μg/mL). After inoculation, the plates were incubated at 42 °C in microaerophilic atmosphere for 24 hours and then screened. *C. jejuni* strain NCTC 11351 was used as control.

### 2.4. Sequencing

*Campylobacter* strains resistant to nalidixic acid and/or ciprofloxacin were sequenced to evaluate any Quinolone Resistance–Determining Region (QRDR) mutation of gyrA gene. The sequencing was performed as suggested by Zirnstein [14] using Big Dye Terminator v3.1 Cycle Sequencing Kit (Applied Biosystems) according to the manufacturer instructions with the Thermal Cycler GenAmp 9700 (Applied Biosystems). The product was purified by Agencourt CleanSEQ and Dye-Terminator Removal (Agencourt Bioscience Corporation, Madison, WI, USA). Sequencing was carried out with the Avant Genetic Analyzer 3100 (Applied Biosystems).

### 2.5. Pulsed Field Gel Electrophoresis (PFGE)

PFGE was performed according to the instructions of the 2009 U.S. PulseNet protocol for *Campylobacter*. Bacteria, previously identified by PCR, were subcultured onto Columbia agar and embedded in agarose blocks (Seakem Gold agarose, Lonza, Rockland, ME, USA). The blocks were then lysed, washed, digested with SmaI restriction enzyme (Promega, Milan, Italy) and subjected to pulsed-field electrophoresis in 1% agarose gel (Seakem Gold agarose, Lonza) for 18 h (Chef Mapper II, Biorad Laboratories, Hercules, CA, USA). *Salmonella* serovar *Branderup* H9812 was used as standard molecular weight size. After electrophoresis run, the gel was stained with Sybr Safe DNA gel stain (Invitrogen) and photographed at transilluminator (Alpha Innotech). The image analysis was performed using the program Bionumerics v. 6.6 (Applied Maths NV, Sint-Martens-Latem, Belgium). Pair comparisons and cluster analyses were carried out using the Dice correlation coefficient and the unweighted pair group mathematical average (UPGMA) clustering algorithm. The optimization tolerance was set at 2.5% and the position tolerance for band analysis was set at 1%.

### 2.6. DNA Microarray

Bacterial DNA was labelled using the Bioprime DNA labelling system kit (Invitrogen Life Technologies, Milano, Italy) as described previously [15]. The labelling efficiency and the percentage of dye incorporation were quantified by scanning the DNA samples at wavelengths from 200 up to 700 nm using a NanoDrop Spectrophotometer (NanoDrop Products, ThermoScientific, Wilmington, DE, USA) and analyzing data with the internet–based Percent Incorporation Calculator (http://www.pangloss.com/seidel/Protocols/percent_inc.html).

Virulence gene profiles were determined using a DNA microarray composed of 70-m oligonucleotide probes targeting virulence associated genes of *Campylobacter* species [16]. Hybridizations were performed as suggested by Bruant [15]. An amount of 500 ng of labelled DNA was dried under vacuum in a rotary desiccator (Savant SpeedVac^®^, ArrayIt, Holbrook, NY, USA) and resuspended in a hybridization buffer (Dig Ease Buffer, Roche Diagnostics spa, Milan, Italy). Before hybridization, microarrays were pre-hybridized for at least one hour at 42 °C in a pre-heated pre-hybridization solution containing 5X SSC, 0.1% SDS (Sigma Aldrich spa, Milan, Italy) and 1.0% BSA (Sigma Aldrich spa). After pre-hybridization, the microarrays were hybridized mixing a solution of Dig Easy Hyb buffer (Roche Diagnostics), Bakers Yeast tRNA (10 mg/ml) (Sigma Aldrich spa), Sonicated Salmon Sperm DNA (10 mg/mL) (Sigma Aldrich spa) with previously denatured labelled DNA. Microarrays were hybridized overnight at 42 °C in a SlideBooster (Advalytix, ABI, Milan, Italy). After hybridization, the slides were washed with increasing stringency washes (1X SSC, 0.1% SDS preheated to 42 °C; 1X SSC and 0.1X SSC at room temperature). Microarray slides were scanned using a ScanArray Lite fluorescent microarray analysis system (Perkin Elmer, Milan, Italy) at excitation wavelengths of 532 nm (Cy3) and 635 (Cy5) and then analysed with the ScanArray Gx software (Perkin Elmer). Images were examined using the QuantArray software version 3.1 (Packard Bioscience, Boston, MA, USA).

The data were normalized as described previously [17]. For each subarray, after subtraction of local background intensity, the median value for each set of triplicate spotted probes was divided by the empty signal and then logarithmically transformed. The data file was then elaborated with Cluster software [18,19]. Strains were clustered by hierarchical clustering using the algorithm Centered Pearson Correlation Distance and Pairwise Maximum Linkage method. For visualization of the elaborated data, Java TreeView, an Open Source program, was utilised [18–20].

## 3. Results and Discussion

Multiplex PCR identified 62.75% of the isolates as *C. jejuni* and 37.24% as *C. coli* (Table 1). In this study the antimicrobial resistance and two methods (PFGE and microarray) for genome analysis of *C. jejuni* and C. *coli* strains were evaluated.

**Figure 1.**
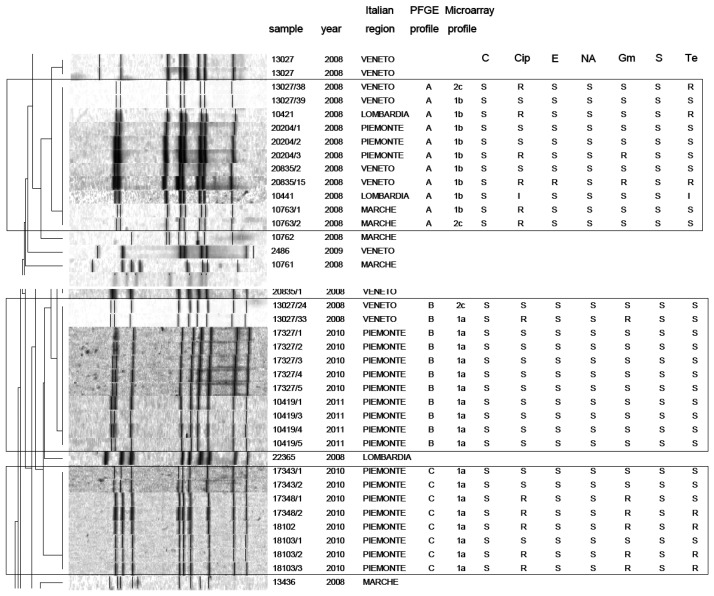
Clustering of PFGE profiles combined with microarray results and antimicrobial resistance profile.

The antibiotic resistance profiles of the isolates are shown in Table 2. In particular, 100 (68.97%) isolates were resistant to at least one antibiotic, whereas the remaining strains (31.03%) were susceptible to all antibiotics tested. The highest levels of resistance were found for ciprofloxacin (62.76%), tetracycline (55.86%) and nalidixic acid (55.17%). In contrast, only 19 (13.10%) strains were resistant to erythromycin, 7 (4.83%) strains to streptomycin and only one (0.69%) isolate to chloramphenicol. *Campylobacter* resistance to ciprofloxacin, nalidixic acid and tetracycline was higher than the respective means at European level (50%, 51% and 37%, respectively) and lower than those reported for Italy in the EFSA Report of 2008 [6]. All *Campylobacter* isolates were found susceptible to gentamicin and 144/145 strains were susceptible to chloramphenicol (Figure 1). Antibiotics resistance was significantly more frequent for *C. coli* when compared to *C. jejuni* only for erytromycin, nalidixic acid and tetracycline (p < 0.05, χ2 test) (Figure 1).

Regarding the distribution patterns of antimicrobial resistance, 5 (3.45%) strains were resistant to only one antibiotic, while 95 (65.52%) strains showed multiple drug resistance to at least two classes of antibiotics (Table 3), differently from some data reported in literature [21–23].

The most common multiple resistance patterns were ciprofloxacin-nalidixic acid-tetracycline (50.52%), ciprofloxacin-erythromycin-nalidixic acid-tetracycline (14.73%), ciprofloxacin-tetracycline (12.63%) and ciprofloxacin-nalidixic acid (9.47%) (Table 3).

Our study revealed that 83 *Campylobacter* strains resistant to ciprofloxacin and/or nalidixic acid presented the mutation T86-I, while only one resistant strain showed no mutation. Probably this strain could have developed a resistance mechanism depending on other characteristics such as changes to the efflux pump [24]. These data confirm the European trend of an increase in *Campylobacter* antibiotic resistance [6] and the study of mutations involved in resistance acquisition process seem to reflect the clonality of the most common mutation T86-I.

**Figure 2.**
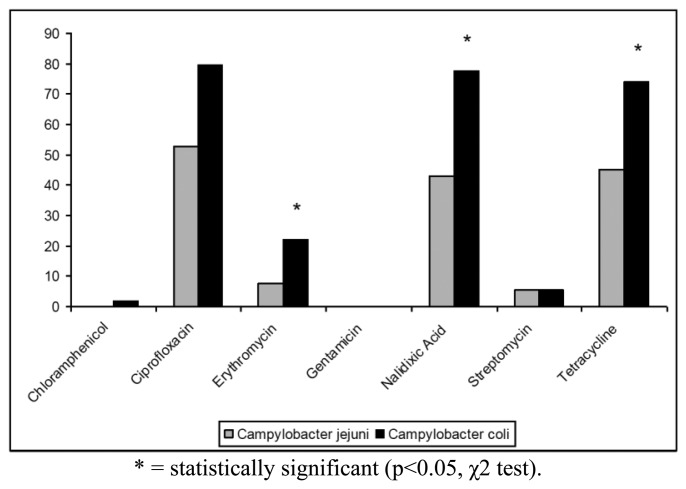
Prevalence (%) of antibiotic resistant *C. jejuni* and *C. coli* strains.

PFGE analysis of *Campylobacter* strains yielded 86 PFGE profiles (isolates clustering ≥95% similarity). Among them, 47 *C. jejuni* and 39 *C. coli* unique macrorestriction profiles were identified. Clustering of *C. jejuni* showed three main clonal groups, A, B, C (Figure 2). Cluster A consisted of 11 isolates from chicken faeces and carcasses from Regions of Northern Italy (Lombardia, Veneto, Piemonte) and from one region of Central Italy (Marche). Cluster B comprised a group of 11 isolates from raw milk and chicken carcasses from Piemonte and Veneto Regions. Cluster C included 8 strains from raw milk from Piemonte Region. PFGE analysis of *C. coli* yielded many micro-groups consisting of a limited number of isolates. Interesting was the finding that all groups were constituted of *Campylobacter* isolated from regions of Northern Italy, providing precious information not only to confirm the geographical relatedness of the strains, but also for future monitoring of *Campylobacter* movements along the national territory. Moreover our results confirm bibliographical data about the high genetic diversity related with this microorganism [25] and its weak clonal population structure. Despite its high discriminatory power, PFGE still remains a difficult technique to standardise and data deriving from band analysis are not always easy to compare among different laboratories [26].

**Figure 3.**
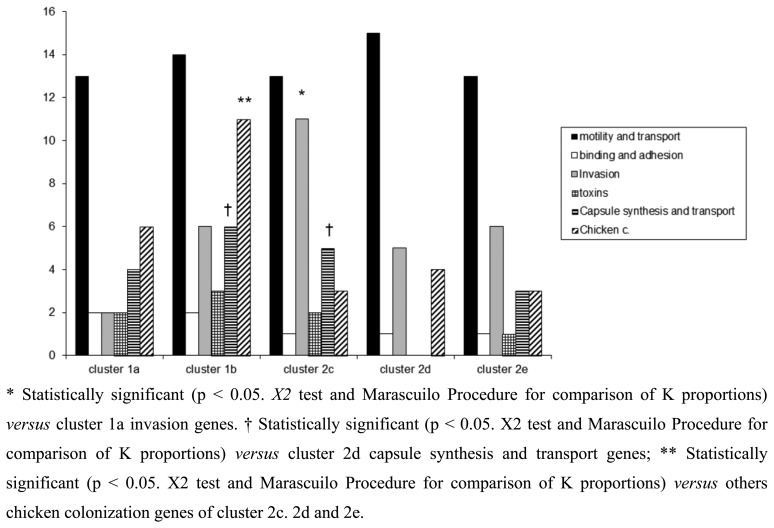
Hybridization patterns for *Campylobacter*. The columns represent the class of genes within each cluster and the heights indicate the number of the present genes.

In this study, a hierarchical clustering analysis using microarray data to identify similarities among the isolates was also performed. The microarray-based comparative genomic hybridizations data were generated using an oligonucleotide array which was evaluated for its ability to discriminate between present/absent virulence genes associated with campylobacteriosis infection. Five significant clusters were obtained (1a, 1b, 2c, 2d, 2e) and data regarding strains geographical origins, matrices and resistant/sensitive patterns toward fluoroquinolones are shown in Table 4. The virulence genes examined are listed in Table 5 and their presence within the clusters is shown in Figure 3. Strain origins and sources had no effect on clustering. *C. jejuni* strains were present in the first two clusters, 1a and 1b, while *C. coli* were present in clusters 2d and 2e and both constituted cluster 2c (Table 4).

The microarray analysis showed a different level of discrimination between clusters based on different virulence gene targets as shown in Figure 3. Virulence genes were present in almost all clusters, with the exception of cluster 2d, in which no toxins, capsule synthesis and transport genes were detected. Moreover a statistically significant presence of genes associated to invasion, capsule synthesis, transport and chicken colonization was observed in clusters 1b and 2c, as presented in Figure 3.

Of the virulence motility genes analysed, 86.6% appeared to be common to all strains. This finding was expected since these genes mainly encode for factors playing a fundamental role in the early phases of infection. Instead virulence genes belonging to invasion, capsule synthesis, transport and chicken colonization were found highly divergent among the clusters, indicating how the selective environmental pressures can drive evolutionary changes in order to differentiate *Campylobacter* strains.

With regards to some important adhesion and binding factors, it was possible to notice the presence of genes coding for the protein binding Peb1 [27] and for the outer membrane protein CadF [28] only in *C. jejuni* belonging to clusters 1a and 1b. Instead genes coding for cytolethal distending toxins (cdtA, cdtB and cdtC) were present only in *C. jejuni* cluster 1b, suggesting a greater potential of invasion for this group of bacteria [16]. In the cluster 2c, positive signals for the presence of genes involved in the biosynthesis of the inner and outer core of LOS were obtained. Another interesting finding was the presence of neuA gene in the clusters 2d and 2e, grouping only *C. coli* strains. This gene is involved in the pathogenesis of GBS being essential for the formation of structures similar to the LOS and human gangliosides [29]. Recently, *C. coli* strains were identified in faeces of patients with GBS [30,31] and the presence of the epitope-NeuAc, crucial for molecular mimicry, was reported [32]. With the exception of *C. jejuni* cluster 2d, the other clusters showed positive signals for genes implicated in capsule synthesis. The last class of genes analysed in this study, *i.e.*, genes involved in the colonization of the gastrointestinal tract of the chicken, were present in all groups of *Campylobacter*, with a significant prevalence for *C. jejuni* strains of cluster 1b. The prevalence of LOS genes and invasion antigen CiaB in the cluster 2c reinforce the idea about the existence of differences in pathogenetic mechanisms among the strains, with the probable emergence of new and more aggressive pathotypes. This cluster grouped the small number of *Campylobacter* strains isolated from human faeces.

On the other hand, the correlation between PFGE and microarray results is very interesting. In particular a close correspondence between *Campylobacter* clusters 1a and 1b (microarray) and clusters B-C and A (PFGE) was noticed. Nevertheless, the two techniques placed the remaining strains in different groups. This is not surprising, since the two techniques process genomes differently.

## 4. Conclusions

The combination of two molecular methods (microarray and PFGE) seems to confirm the genetic similarity of strains clustered from regions of northern Italy (Piemonte, Veneto and Lombardia) and to establish a possible correlation. The mechanisms that induce genetic diversity in *Campylobacter*, however, still remains poorly understood. It is well known that *C. jejuni* is naturally competent and this aspect, combined with its high rate of recombination, can contribute to its genetic diversity, as shown by the horizontal intra-species and inter-species genetic exchange in *C. jejuni* [46]. Molecular typing holds a significant role in epidemiological investigations and surveillance networks, improving the ability to detect outbreaks, thus representing a tool to trace back sources and pathogens throughout the food chain. Its use offers opportunities to better understand epidemiology, ecology and population genetics of food-borne pathogens. However further strategies are needed to monitor and control bacterial infections in food production and new guidelines are required for limiting the use of chemicals only to those cases they are strictly necessary. Moreover constant monitoring of the antibiotic resistance development from enteropathogenic bacteria is essential to understand the trend and to plan efficacious intervention strategies.

## Figures and Tables

**Table 1.** Multiplex PCR results.

**Type**	**N°** ***Campylobacter*** **(%)**	**Matrix (%)**				

		**Raw** **Milk**	**Chicken** **Faeces**	**Chicken** **Carcasses**	**Cattle** **Faeces**	**Human** **Faeces**
*Campylobacter* *jejuni*	91 (62.75)	24 (26.37)	21 (23.07)	41 (45.05)	1 (1.09)	4 (4.39)
*Campylobacter* *coli*	54 (37.24)	1 (1.85)	22 (40.74)	31 (57.4)	-	-

**Table 2.** Antimicrobial resistance profiles.

	**Resistance profile** **N° strains (%)**						

	**C**	**Cip**	**E**	**NA**	**Gm**	**S**	**Te**
Resistant	1 (0.69)	91 (62.76)	19 (13.10)	80 (55.17)	0	7 (4.83)	81 (55.86)
Intermediate	0	6 (4.14)	4 (2.76)	0	0	0	4 (2.76)
Sensitive	144 (99.31)	48 (33.10)	122 (84.14)	65 (44.83)	145 (100)	138 (95.17)	60 (41.38)

C = chloramphenicol; Cip = Ciproflox0acin; E = erythromycin; NA = nalidixic acid; Gm = gentamicin; S = streptomycin; Te = tetracycline.

**Table 3.** Multiple resistance patterns.

**Multiples Resistance**	**N° Antibiotics**	**N° Strains (%)**
CipNaESTe	5	3 (3.16)
		1 (1.05)
CCipENaTe		
CipENaTe	4	14 (14.73)
CipNaSTe		2 (2.10)
CipNaTe	3	48 (50.52)
CipETe		3 (3.16)
ENaS		1 (1.05)
CipNaS		1 (1.05)
CipENa		1 (1.05)
CipNa	2	9 (9.47)
		12 (12.63)

Tot. multiresistent strains		95 (65.52)

C = chloramphenicol; Cip = ciproflox0acin; E = erythromycin; NA = nalidixic acid; Gm = gentamicin; S = streptomycin; Te = tetracycline.

**Table 4.** Microarray clustering results.

**Cluster**	***C.jejuni***	***C.coli***	**Resistent to Fluoroquinolones**	**Sensitive to Fluoroquinolones**	**Matrix**	**Italian Regions**
1 a	23	-	7 (30.43%)	16 (69.56%)	18 RAW (78.26%) 5 CC (21.74%)	(69.56%) Piemonte (30.43%) Veneto
1 b	47	-	24 (51.06%)	23 (48.93%)	21 CC (44.68%) 18 CF (38.29%) 6 RAW (12.76%) 1 BF (2.12%) 1 DH (2.12%)	(36.17%) Piemonte (25.53%) Veneto (13.83%) Lombardia (9.58%) Marche (12.76%) Campania (2.12%) Sicilia
2 c	21	5	5 (100%) *C.coli* 18 (79.23%) *C.jejuni*	3 (12.5%) *C.jejuni*	3 DH (14.28%) 18 CC (85.71%) 5 CF (19.23%) (*C.coli*)	(3.84%) Piemonte (57.69%) Veneto (7.69%) Lombardia (30.77%) Marche
2 d	-	42	34 (80.95%)	8 (19.04%)	48 CF (97.95%) 1 RAW (2.04%)	(23.80%) Piemonte (7.46%) Veneto (35.42%) Lombardia (11.90%) Marche (4.76%) Abruzzo (7.14%) Molise (9.52%) Campania
2 e	-	7	7 (100%)	-	4 CC (57.14%) 3CF (42.85%)	(28.57%) Piemonte (42.85%) Lombardia (28.57%) Marche

RAW. raw milk; CC. chicken carcasses; CF. chicken faeces; BF. cattle faeces; DH. faeces of diarrhoeic patients.

**Table 5.** List of more representative bacterial virulence genes analysed.

	**Genes**	**Function**	**Reference**
Motility	flaG; flaB; flaA; flaD	flagellin proteins	[33–35]
	flgG2	flagellar basal-body rod protein	[33]
	flgK	flagellar hook-associated protein	[33]
	flhB; flhA	flagellar biosynthesis protein FlhB;FlhA	[33]
	fliA	flagellar biosynthesis sigma factor	[35]
	fliI	flagellum –specific ATP synthase	[33]
	fliM; fliG; fliN	flagellar motor switch protein	[33]
	mot A.B	flagellar motor proteins	[33]
Adhesion	cadF	fibronectin binding outer membrane protein	[28]
	peb1	periplasmic binding protein	[27]
	porA	major outer membrane protein	[33]
	jlpA	surface-exposed lipoprotein	[36]
Invasion	LOS (waaF; waaC; wlaN; cst; neuB1; neuA1; waaV; waaD; waaM; rfaE/hldE)	mimicry with GM1 and GD1gangliosides leading to GBS to Guillain–Barrè syndrometo to Guillain;Barrè syndrome	[37]	
	CiaB	*Campylobacter* invasive antigens	[38,39]	
	CPS	capsular polysaccharide	[40]	
	cadF	fibronectin binding protein	[41]	
Toxins	cdtA.B.C	cytolethal distending toxins	[42,43]	
Capsule synthesis	kpsS;kpsF;kpsM;kpsE;kpsT;kpsD	capsule polysaccharide export protein	[35]	
	gmhA2	phosphoheptose isomerase	[33,44]	
	Cj1418c	hypothetical protein	[33]	
	Cj1420c	methyltransferase	[33]	
Chicken colonization	rpoN	transcription of flagellar genes	[44]	
	cheY	chemotaxis protein	[33]	
	pglH, wlaJ/pglE, pglF	Protein glycosylation	[45]	
	livj	Probable transport system periplasmic binding protein	[45]	
	pta	Probable phosphate acetyltransferase	[45]	
	docB	Probable methyl;accepting chemotaxis domain singal transduction protein	[45]	
	Cj0903c	Probable amino acid transport protein	[45]	
	Cj0618c	Unknown identity	[45]	
	Cj0454c	Probable membrane protein	[45]	
	Cj0456c	Unknown identity	[45]	
	aas	Probable 2-acylglycerophosphoethanolamine acyltransferase/acyl-acyl carrier protein synthetase	[45]	
